# A Diterpenoid, 14-Deoxy-11, 12-Didehydroandrographolide, in *Andrographis paniculata* Reduces Steatohepatitis and Liver Injury in Mice Fed a High-Fat and High-Cholesterol Diet

**DOI:** 10.3390/nu12020523

**Published:** 2020-02-18

**Authors:** Yun-Ta Liu, Haw-Wen Chen, Chong-Kuei Lii, Jia-Hua Jhuang, Chin-Shiu Huang, Mei-Ling Li, Hsien-Tsung Yao

**Affiliations:** 1 Department of Nutrition, China Medical University, 91 Hsueh-shih Road, Taichung 404, Taiwan; hhh12324@msn.com (Y.-T.L.); chenhw@mail.cmu.edu.tw (H.-W.C.); cklii@mail.cmu.edu.tw (C.-K.L.); angel2088250@gmail.com (J.-H.J.); u104059001@cmu.edu.tw (M.-L.L.); 2 Department of Health and Nutrition Biotechnology, Asia University, Taichung 413, Taiwan; cshuang@asia.edu.tw

**Keywords:** *Andrographis paniculata*, 14-deoxy-11,12-didehydroandrographolide (deAND), NLRP3 inflammasome, liver injury, steatohepatitis

## Abstract

14-Deoxy-11,12-didehydroandrographolide (deAND), a diterpenoid in *Andrographis paniculata* (Burm. f.) Nees, acts as a bioactive phytonutrient that can treat many diseases. To investigate the protective effects of deAND on reducing fatty liver disease, male mice were fed a high-fat and high-cholesterol (HFHC) diet without or with 0.05% and 0.1% deAND supplementation. Cholesterol accumulation, antioxidant, and anti-inflammatory activities in liver and liver injury were evaluated after deAND treatment. The results show that deAND treatment for seven weeks reduced plasma alanine aminotransferase activity and lowered hepatic cholesterol accumulation, tumor nuclear factor-α, and histological lesions. The 0.1% deAND treatment reduced HFHC diet-induced apoptosis by lowering the caspase 3/pro-caspase 3 ratio. After 11 weeks of deAND treatment, increased NOD-like receptor protein 3 (NLRP3), capase-1, and interleukin-1β protein levels in liver were suppressed by deAND treatment. In addition, nuclear factor erythroid 2-related factor 2 (Nrf2) mRNA expression, heme oxygenase-1 protein expression, and the activities of glutathione peroxidase and glutathione reductase were increased in mice fed the HFHC diet. However, those activities of antioxidant enzymes or proteins were also upregulated by 0.1% deAND treatment. Furthermore, deAND treatment tended to lower hepatic lipid peroxides. Finally, deAND treatment reversed the depletion of hepatic glutamate level induced by the HFHC diet. These results indicate that deAND may ameliorate HFHC diet-induced steatohepatitis and liver injury by increasing antioxidant and anti-inflammatory activities.

## 1. Introduction

Nonalcoholic fatty liver disease (NAFLD) is the most common liver disease, encompassing a range of illnesses, from simple steatosis to nonalcoholic steatohepatitis. Its estimated global prevalence is about 25% [1]. Oxidative stress and inflammation in the liver are believed to play an important role in the development and progression of this disease [2]. Recent studies have demonstrated that accumulations of high cholesterol and cholesterol crystals in the livers increased cholesterol-induced NOD-like receptor protein 3 (NLRP3) inflammasome activation and thus resulting in an increase in caspase 1-mediated interleukin (IL)-1β protein release [3]. 

Animal models of diet-induced steatohepatitis such as methionine and choline-deficient diet or high-fat and high-cholesterol (HFHC) diet can increase hepatic fat accumulation, inflammation, and liver injury, but these two models may also reduce body weight [4,5]. Mice fed a HFHC diet develop hypercholesterolemia and accumulate cholesterol in the liver without the occurrence of obesity and insulin resistance [3,6,7]. Although this animal model may still be controversial for patients with nonalcoholic steatohepatitis (NASH), it can be used as an experimental model for evaluating the testing compound on steatohepatitis and liver damage. In our previous study, mice fed a HFHC diet for six weeks showed increased hepatic cholesterol accumulation, NOD-like receptor protein 3 (NLRP3) inflammasome activation, and liver injury, as indicated by an increase in plasma alanine aminotransferase (ALT) activity and the presence of histological lesions in the liver [8]. Long-term HFHC diet feeding (>6 weeks) has been shown to increase oxidative stress, inflammation, and fibrosis in the livers of mice [9]. Mice fed a HFHC diet with active components of functional foods (e.g., green tea polyphenols or freshwater clam extract) have been demonstrated to prevent steatosis, inflammation, and liver injury [8,10].

Phytonutrients are chemicals produced by plants with specific biological activities that can improve human health. Important bioactive phytonutrients include polyphenols, flavonoids, terpenoids, carotenoids, limonoids, glucosinolates, phytoestrogens, phytosterols, and anthocyanins, etc. [11]. Recently, phytonutrients have received increased attention in studies on the prevention of many diseases, including NASH [10,12]. Unlike clinical drugs, phytonutrients are natural products that possess relatively few or no side effects when they are used for therapy [11]. *Andrographis paniculata* (Burm. f.) Nees is a traditional medicine used in Chinese, Indian, and Thai remedies that are commonly used to treat infections, colds, and diarrhea [13]. A. *paniculata* and its related products are also used as functional foods in Taiwan. Terpenoids are the most attractive phytonutrients of *A. paniculata*, and a number of diterpenoids have also been identified, including andrographolide (AND), 14-deoxy-11,12-didehydroandrographolide (deAND), neoandrographolide, 14-acetylandrographolide, and 14-deoxyandropholide [14]. Of these, AND is the most abundant terpenoid in *A. paniculata* and has been reported to have many biological functions, including liver-protective effects probably resulting from increases in antioxidant and anti-inflammatory activities [15,16,17,18]. deAND, the second-most abundant diterpenoid in *A. paniculata*, is present in a comparative concentration to AND in the leaves of the plant (~17.4 mg/g) [19]. Although relatively less information is known about its biological functions compared with AND, deAND exerts no toxicity [20] and has higher oral bioavailability than that of AND [15,21]. In previous studies, the anti-cancer, anti-virus, anti-inflammation, and cardiovascular protective effects of deAND have been reported [20,22,23,24,25,26]. 

Because AND has been demonstrated to have liver-protective effects [16,17,18,19], it is possible that deAND may be an effective agent on treating liver diseases. This study was the first to investigate the hepatoprotective effects of deAND in a HFHC diet-induced steatohepatitis and liver injury in mice. The potential effects of deAND on antioxidant and anti-inflammatory activities in liver were determined.

## 2. Materials and Methods

### 2.1. Materials

**Figure 1:**
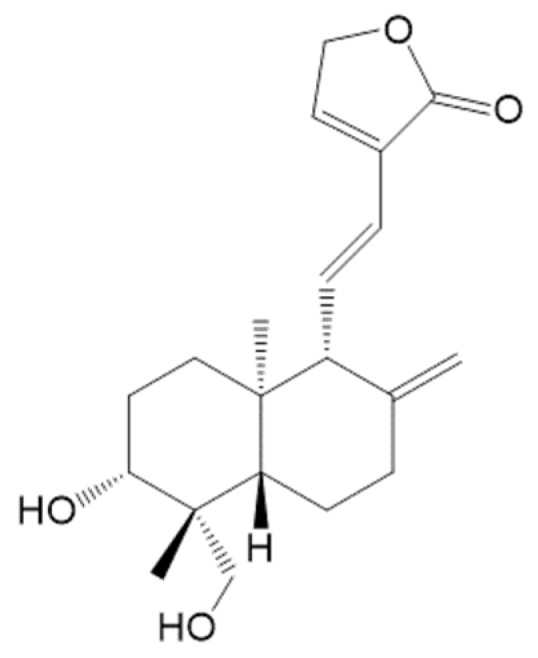
Chemical structure of 14-Deoxy-11,12-didehydroandrographolide (deAND).

Nicotinamide-adenine dinucleotide phosphate (NADPH), glutathione (GSH), 1-chloro-2,4-dinitrobenzene, 1,1,3,3-tetraethoxypropan, thiobarbituric acid, and heparin were obtained from Sigma Aldrich (St. Louis, MO, USA). All other chemicals and reagents were of analytical grade and were obtained commercially. deAND (Figure 1) was obtained according to a previously described method [21]. In brief, dried *A. paniculata* was ground into a fine powder and extracted by 95% ethanol (1:5; w/v) with gentle stirring at room temperature for 24 h. The resulting filtrate was concentrated under a rotatory evaporator and then fractionated between H2O and ethyl acetate (EA) (1:1, v/v). The EA layer was concentrated, and the resulting residue was then mixed with an equal volume of Silica gel (70–230 mesh) and allowed to evaporate until dry. This EA extract was then separated with different gradient solvent systems in Silica gel, and the resulting solvent was crystallized. The crystals were dissolved in methanol and separated by a Sephadex LH-20 column and gel filtration, and, finally, they underwent crystallization again. Chemical identity was confirmed by high performance liquid chromatography (HPLC)/mass spectrometers (MS) and ^1^H-NMR. The purity of the deAND used was >98%. 

### 2.2. Animal Studies

In Experiment 1, the effects of deAND on the hepatic fat content, oxidative stress, and inflammation in the livers of mice were investigated. Male C57BL/6J mice (six weeks old), obtained from the National Laboratory Animal Center (Taipei, Taiwan), were fed a pelleted diet for one week during the adaptation period and were then randomly divided into four groups with six mice per group, as follows: (1) Control group, (2) HFHC group, (3) HFHC + 0.05% deAND group, and (4) HFHC + 0.1% deAND group. The HFHC group contains high-fat and high-cholesterol in the diet. The compositions of the experimental diets are shown in Table 1. The total calories (Kcal/100 g diet) in the low-fat control group and the HFHC group were 381.7 Kcal/100 g and 494.9 Kcal/100 g, respectively. The vitamin and mineral mixtures (AIN 93) were purchased from ICN Biochemicals (Costa Mesa, CA). Mice were fed the experimental diets for seven weeks. The daily oral dose of deAND for the 0.1% deAND group was approximately 100 mg/kg BW. The initial average animal body weight was 23.2 ± 1.4 g. Mice were housed in plastic cages in a room kept at 23 ± 1 °C and 60 ± 5% relative humidity with a 12 h light-dark cycle. Food and drinking water were available ad libitum. Food intake was measured every week. At the end of the study, feces were collected for three consecutive days. Then, the mice were fasted overnight and sacrificed by cardiac puncture after carbon dioxide asphyxiation. Heparin was used as the anticoagulant and plasma was separated from the blood by centrifugation (1750× *g*) at 4 °C for 20 min. The concentrations of plasma alanine aminotransferase (ALT), aspartate aminotransferase (AST), high-sensitive C reactive protein (Hs-CRP), total cholesterol, and triglyceride were measured immediately by an autoanalyzer (DiaSYS Diagnostic system, Germany). The plasma interleukin-1β (IL-1β) concentration was determined with a mouse IL-1β ELISA kit (R&D Systems). Part of each liver sample was excised and fixed in 10% neutral formalin followed by dehydration in ascending grades of alcohol, clearing in xylene, and embedding in paraffin wax. Liver sections (5 μm thickness) were stained with hematoxylin and eosin (H&E) for the histological examination [27]. The other liver samples from each animal were stored at –80 °C.

In Experiment 2, the effects of deAND supplementation in conjunction with a long-term (11 weeks) HFHC feeding on preventing steatohepatitis, and its possible mechanisms for reducing inflammation and oxidative stress were investigated. Male C57BL/6J mice (6 weeks old) were divided into the same four groups as described above with six mice per group and provided low-fat or HFHC diet containing 0.05% or 0.1% deAND. At the end of the experiment, plasma and liver samples were collected using the same procedures as described above. The activities of antioxidant enzymes, nuclear factor erythroid 2-related factor 2 (Nrf-2) mRNA expression, NLRP3 inflammasome activation, and histological examination (H&E and masson’s trichrome-stained) in the liver were determined. This study was approved (2018–140) by the Animal Center Management Committee of China Medical University. The animals were maintained in accordance with the guidelines for the care and use of laboratory animals [28].

### 2.3. Determinations of Fat Contents in the Liver and Feces 

The total lipid content was extracted from the liver and feces with a chloroform/methanol solution (v/v, 2:1) according to the method of Folch et al. [29]. Cholesterol and triglyceride in the solvent extract were emulsified by the addition of Triton x-100 and their concentrations were determined with enzymatic kits (Randox Ltd, Antrim, UK). The total bile acids content in dry feces was extracted with methanol and quantified using an enzymatic kit (Randox Ltd, Antrim, UK).

### 2.4. Determination of Glutathione, Glutathione-Related Enzyme Activities, and Lipid Peroxidation in Liver 

To obtain a 10% (w/v) liver homogenate, 1 g of liver tissue was homogenized with 9 mL of 1.15% KCl. The liver homogenate was then centrifuged at 10,000× *g* for 15 min at 4 °C. The resulting supernatant was used to determine the contents of reduced GSH and lipid peroxides as well as the activities of GSH-related enzymes. The GSH content in the liver homogenate was determined by high performance liquid chromatography (HPLC)/mass spectrometer (MS) [30]. The thiobarbituric acid-reactive substance (TBARS) value, as an index of the lipid peroxide level, in tissue homogenate was determined according to the method of Uehiyama and Mihara [31]. Malondialdehyde (MDA) was used as a standard to calculate the TBARS value. GSH peroxidase, GSH reductase, and GSH-S-transferase activities were determined by spectrophotometer according to the method reported previously [32].

### 2.5. Western Blot Analysis

The Western blot analysis was performed as described previously [33]. The liver homogenates of each group with equal amounts of protein were separated by SDS-PAGE and transferred to polyvinylidene difluoride membranes. After blocking of the nonspecific binding sites with 5% non-fat dry milk in 15 mM Tris/150 mM NaCl buffer (pH 7.4), the membranes were hybridized with antibodies against anti-NLRP3 (#15101, Cell Signaling Technology, Danvers, MA, USA), anti-caspase-1 (#3019, BioVision Inc, Milpitas, CA, USA), anti-IL-1β (ab9722, Abcam, Cambridge, UK), anti-caspase 3 (SC-56053, Santa Cruz Biotechnology, CA, USA), anti-pi class GSH-S-transferase (PGST) (610719, BD Biosciences, San Jose, CA, USA), anti-heme oxygenase-1 (HO-1) (374090, Merck Millipore, Billerica, MA, USA), and GAPDH (GTX100118 GeneTex Inc, Irvine, CA, USA).

### 2.6. Quntitative Real-Time Polymerase Chain Reaction (Q-PCR) Analysis

The total RNA content (1 μg) was extracted from homogenized liver tissue using a TRIZOL reagent (Invitrogen, Carlsbad, CA, USA), according to the manufacturer’s instructions, and reverse-transcribed into first-strand cDNA by using 200 units of MMLV-RT (Promega). The total volume of incubation was 20 μL. For real-time PCR, a SYBR system with self-designed primers and 12.5 ng cDNA was used. The self-designed primers are shown in Table 2. Amplification was performed using 40 cycles of 2 steps (95 °C for 15 s and 60 °C for 1 min) on a Bio-rad CFX connect real-time PCR detection system (Hercules, CA, USA) installed with CFX Manager Software, which measures the threshold cycle (C_t_) value. The melting curve was detected after the 40 cycles to approve the representative of the DNA fragments. The level of mRNA expression of each gene was calculated by the 2^−ΔΔCt^ method using GADPH mRNA as the internal control. 

### 2.7. Determination of Glutamate in the Plasma and Liver

An aliquot (50 μL) of plasma or liver homogenate was combined with 100 μL of acetonitrile to precipitate the protein and then centrifuged at 10,000× *g* at 4 °C for 15 min. The supernatant was then analyzed by the HPLC/MS method. The Agilent 1100 series HPLC system was interfaced to an Agilent Mass Selective Detector (MSD) equipped with an electrospray ionization source. An Agilent poroshell 120 Hillic column (2.7 μm, 3.0 mm × 100 mm i.d.) was used to determine the glutamate content. The column temperature was set at 25 °C. Mobile phase A was acetonitrile and mobile phase B was 5% water. A gradient system with the following mobile phase composition was used to separate the glutamate: 95% A to 70% A (0–1 min), 70% A to 50% A (1–6 min), 50% A to 10% A (6–8 min), 10% A to 95% A (8–10 min), and 95% A (10–20 min). The flow rate was 0.3 mL/min, the retention time of the glutamate was 7.7 min. The injection volume was 3 μL. The MS data were acquired via a selected ion monitoring set at 146 (m/z) with a negative ion mode and then the peak area was measured. The calibration standards of the glutamate were prepared by serial dilution of the stock solution of the glutamate with water.

### 2.8. Statistical Analysis

Statistical differences among the groups was determine by one-way ANOVA (SAS Institute, Cary, NC, USA). Differences were considered to be significant at *p* < 0.05, as determined by independent-sample *t*-tests. 

## 3. Results

### 3.1. Plasma Biochemical Parameters 

In Experiment 1, mice fed the HFHC diet increased (*p* < 0.05) plasma total cholesterol concentration when compared with the low-fat control group (Table 3). However, plasma triglyceride concentration was lower (*p* < 0.05) than that of the control group. This observation has also been observed in previous studies [6,8,34], as very low density lipoprotein (VLDL) secretion may have been impaired after feeding with the HFHC diet [34]. Mice fed a HFHC diet with 0.05% or 0.1% deAND supplementation had a reduced (*p* < 0.05) total plasma cholesterol concentration with no change in plasma triglyceride concentration when compared with the animals fed an HFHC diet. In addition, higher (*p* < 0.05) plasma concentrations of ALT, AST, and high-sensitive C reactive proteins (Hs-CRPs) were found in mice fed the HFHC diet compared with those fed the low-fat control diet. The deAND treatment caused a significant reduction (*p* < 0.05) in the activities of plasma ALT and AST, indicating that deAND treatment could lower liver damage. No significant differences (*p* > 0.05) in plasma triglyceride or IL-1β concentrations were observed among the HFHC groups. In this study, mice fed the HFHC diet reduced (*p* < 0.05) body weight (Control group: 24.8 ± 1.2 g; HFHC group: 18.1 ± 1.4 g; HFHC+0.05% deAND group: 17.7 ± 0.9 g; HFHC+0.1% deAND group: 18.6 ± 1.7 g) and food intake (Control group: 4.0 ± 0.0 g; HFHC group: 2.3 ± 0.2 g; HFHC + 0.05% deAND group: 2.4 ± 0.2 g; HFHC + 0.1% deAND group: 2.5 ± 0.2 g) without affecting relative liver weight when compared with those animals fed the low-fat control diet. Therefore, the daily intakes (w/w, %) of protein (−42.5%) and carbohydrate (−65.9%) were reduced; however, the daily fat intake was increased (+251.4%) in HFHC group compared to low-fat control diet. Thus, in this animal model, the lower daily food intake and protein available may possibly result in a lower body weight gain in mice fed the HFHC diet. In this study, deAND treatment had no effect on body weight, food intake, and relative liver weight in mice fed the HFHC diet (data not shown).

### 3.2. Histological Examination

**Figure 2:**
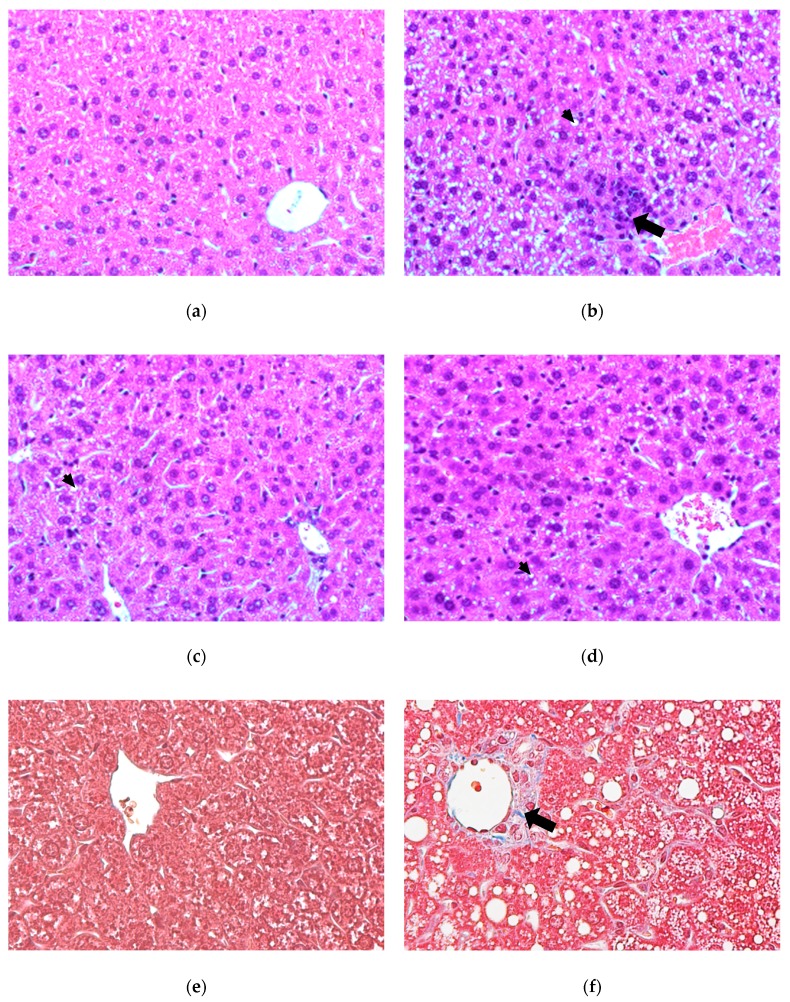

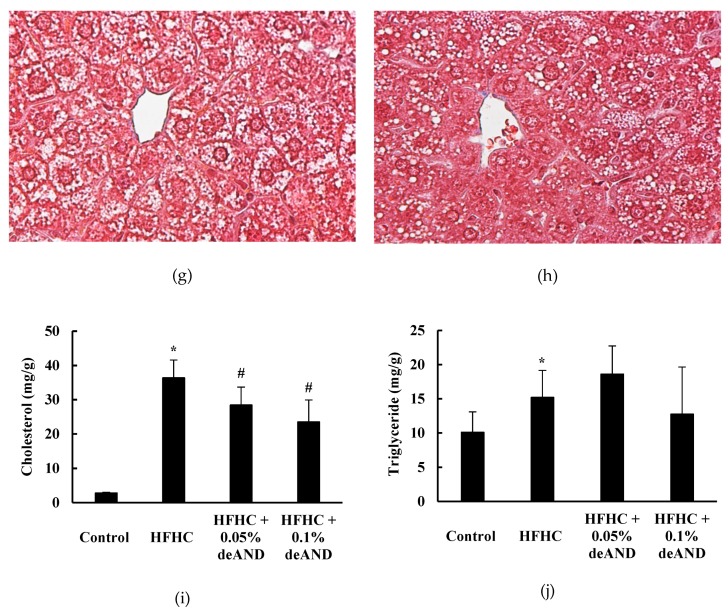
Histopathological examination (H&E stain, 400x) of livers in the control group (**a**), HFHC group (**b**), HFHC + 0.05% deAND group (**c**), and HFHC + 0.1% deAND group (**d**). The small arrow indicates the fat droplets and the large arrow indicates the perivenular inflammatory infiltrates. Normal liver architecture was found in the low-fat control group (**a**). Figure 2e–h shows histopathological examination of liver fibrosis (masson’s trichrome- stain, 400x) in the control group (**e**), HFHC group (**f**), HFHC + 0.05% deAND group (**g**), and HFHC + 0.1% deAND group (**h**). The large arrow in HFHC group indicates the collagen (blue color). Hepatic cholesterol and triglyceride contents are shown in (**i**,**j**). Values are means ± SD. (*n* = 5–6). * Significantly different from the Control group, *p* < 0.05. ^#^ Significantly different from the HFHC group, *p* < 0.05. HFHC, high-fat and high cholesterol. deAND, 14-Deoxy-11,12-didehydroandrographolide.

The histological examination of the hematoxylin and eosin (H&E) stained liver sections is shown in Figure 2a–d. The morphological findings were consistent with those of previous observations, showing that mice fed a HFHC diet had significantly greater accumulation of small lipid droplets in liver cells compared with those fed a low-fat control diet (Figure 2a,b). These small lipid droplets were demonstrated to have abundant cholesterol and/or cholesterol crystals [3]. The HFHC diet feeding also caused significantly perivenular inflammatory infiltrates and ballooning (Figure 2b). These alternations were ameliorated by deAND treatment (Figure 2c,d). However, no obvious difference was found between the two deAND groups. The histological examination of the masson’s trichrome-stained liver sections (Experiment 2) is shown in Figure 2e–h. The stain imparts a blue color to collagen against a red background of hepatocytes. The HFHC diet caused perivenular fibrosis in the liver of mice fed the HFHC diet compared with those fed a low-fat control diet (Figure 2e,f). deAND treatment ameliorated these alternations and no obvious difference was found between the two deAND groups (Figure 2g,h). The histological examination of the liver sections of mice fed the HFHC diet in experimental 2 also showed significant liver hypertrophy and steatohepatitis compared with mice fed the low-fat control diet (Figure S1). In addition, mice fed the HFHC diet had a mild increase (*p* < 0.05) in liver triglyceride content accompanied by a dramatic increase (*p* < 0.05) in liver cholesterol accumulation (Figure 2i,j). deAND treatment reduced (*p* < 0.05) the hepatic cholesterol content in a dose-dependent manner. These results indicate that deAND treatment could reduce cholesterol accumulation and inflammation in the liver.

### 3.3. GSH and GSH-Related Enzyme Activities, Lipid Peroxidation, and Tumor Necrosis Factor-Alpha (TNF)-α Content

As shown in Table 4, mice fed the HFHC diet reduced (*p* < 0.05) GSH level and lowered GSH reductase activity (*p* < 0.05) and GSH peroxidase activity (*p* < 0.1), while hepatic lipid peroxidation (TBARS) and GSH-S-transferase activities were unchanged (*p* > 0.05). However, increased TNF-α level in the liver was observed in mice fed the HFHC diet compared with those animals fed the low-fat control diet (*p* < 0.05). Mice fed the HFHC diet with 0.05% or 0.1% deAND treatment showed no differences (*p* > 0.05) in TBARS or GSH peroxidase and GSH-S-transferase activities. However, the 0.1% deAND group increased (*p* < 0.05) the hepatic GSH and GSH reductase activity and lowered (*p* < 0.05) the TNF-α in the liver when compared with the HFHC group. These results indicate that deAND may exert antioxidant and anti-inflammatory activities in the livers of mice fed a HFHC diet.

### 3.4. Apoptosis Index in Liver

**Figure 3:**
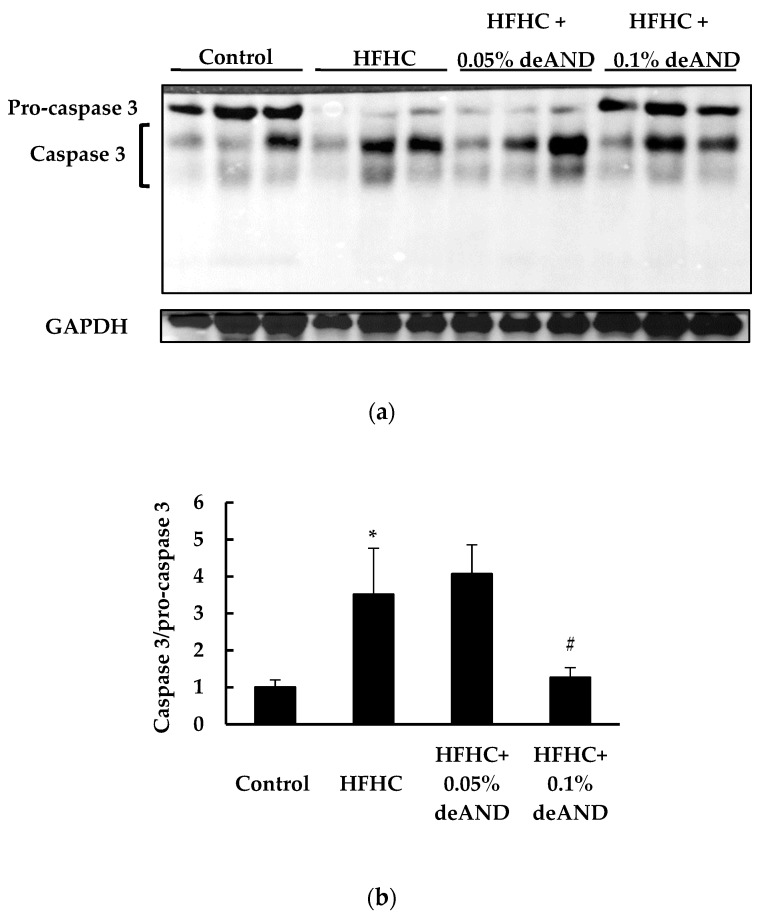
Western blotting analysis of apoptosis index (Caspase 3/pro-caspase 3) in liver (**a**). The data show the effects of deAND supplementation on HFHC diet-induced apoptosis (**b**) in the liver. Glyceraldehyde 3-phosphate dehydrogenase (GADPH) served as the loading control. Active caspase 3, derived from cleavages of pro-caspase 3, was quantitated by the sum of 17 and 20 kd protein bands. The values are given as the mean ± S.D. (*n* = 3). * Significantly different from the Control group, *p* < 0.05. ^#^ Significantly different from the HFHC group, *p* < 0.05.

Figure 3 shows results of the Western blotting analysis of the apoptosis index in the liver. Mice treated with the HFHC diet increased apoptosis due to an increased (*p* < 0.05) caspase 3/pro-caspase 3 ratio [35] compared with that of animals treated with the low-fat control diets. In this study, 0.1% deAND treatment showed a significant decrease in apoptosis (*p* < 0.05) in liver as evidenced by decreased caspase 3/pro-caspase 3 ratio. 

### 3.5. Fecal Cholesterol and Total Bile Acid Contents

**Figure 4:**
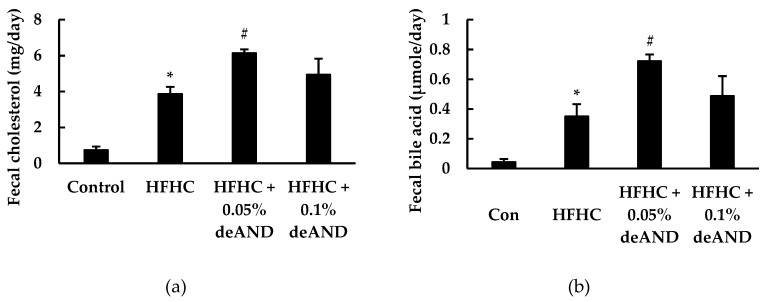
Effects of administrated of deAND on fecal cholesterol (**a**), and total bile acids (**b**) contents in rats. Results are expressed as the mean ± S.D. (*n* = 3). * Significantly different from the control group at *p* < 0.05. ^#^ Significantly different from HFHC group, *p* < 0.05. The feces in each group (six mice in one cage) was collected for three consecutive days and then the fecal cholesterol and bile acids from pooled samples in each group were determined in triplicates.

In this study, mice fed the HFHC increased (*p* < 0.05) fecal excretions of cholesterol and total bile acid contents than in those animals fed the low-fat control diet (Figure 4). deAND treatment was associated with increased (*p* < 0.05) fecal excretions of cholesterol and total bile acids. However, this cholesterol or bile acid-lowering effect of deAND was more significant in mice treated with 0.05% deAND than that of mice treated with 0.1% deAND treatment. 

### 3.6. NLRP3 Inflammasome Activation

**Figure 5:**
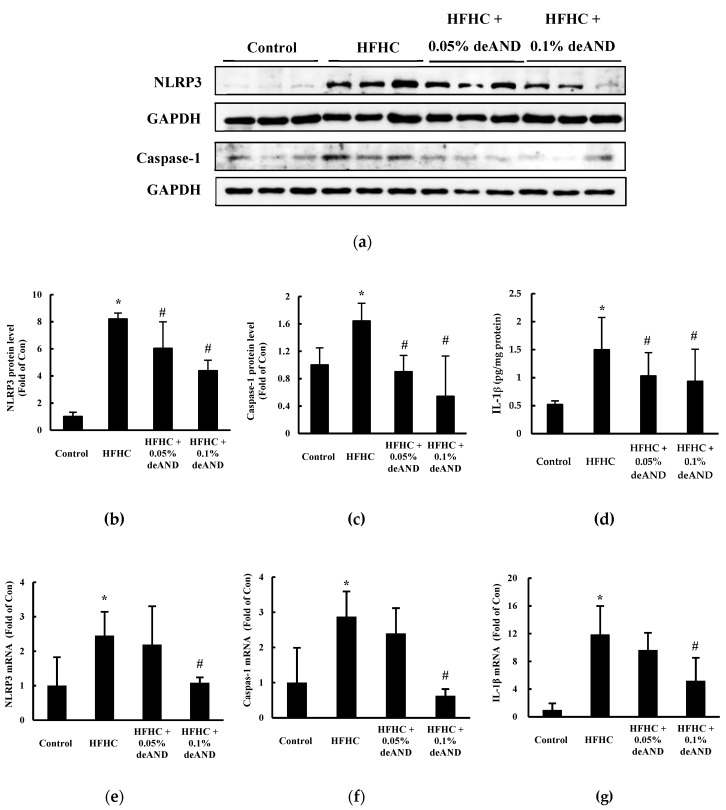
Western blotting analysis of NOD-like receptor protein 3 (NLRP3) and caspase-1 (P10) proteins in the liver (**a**). IL-1β protein level in liver was determined by a commercial kit as described in Material and Methods. The results show that deAND supplementation reduced the expressions of the proteins (*n* = 3 for figure **b** and **c,** and *n* = 6 for **d**) and mRNA (*n* = 6 for figure **e–g**) of NLRP3, caspase-1, and IL-1β in liver. The protein band was quantified by densitometry and mRNA expression level was calculated by the 2 ^-ΔΔCT^ method, respectively. * Significantly different from the Control group at *p* < 0.05. ^#^ Significantly different from the HFHC group, *p* < 0.05.

In Experiment 2, to further investigate the action mechanisms of deAND involved in the anti-inflammatory activity in liver, mice were fed a HFHC diet with a longer feeding period (11 weeks), and then the hepatic NLRP3 inflammasome activation was determined. Immunoblots revealed that higher protein expressions of NLRP3 and activated caspase-1 (p10) were present in HFHC-fed mice than in those animals fed the low-fat control diet (*p* < 0.05; Figure 5a). deAND treatment dose-dependently reduced (*p* < 0.05) NLRP3, caspase-1, and IL-1β protein levels (Figure 5b–d). Similar changes in mRNA expression were observed in mice fed the HFHC diet with deAND treatment as well (Figure 5e–g). In this study, the HFHC diet did not affect body weight gain when compared with the low-fat control diet. deAND treatment had no effect on body weight (data not shown).

### 3.7. Antioxidant Enzyme Activity

**Figure 6:**
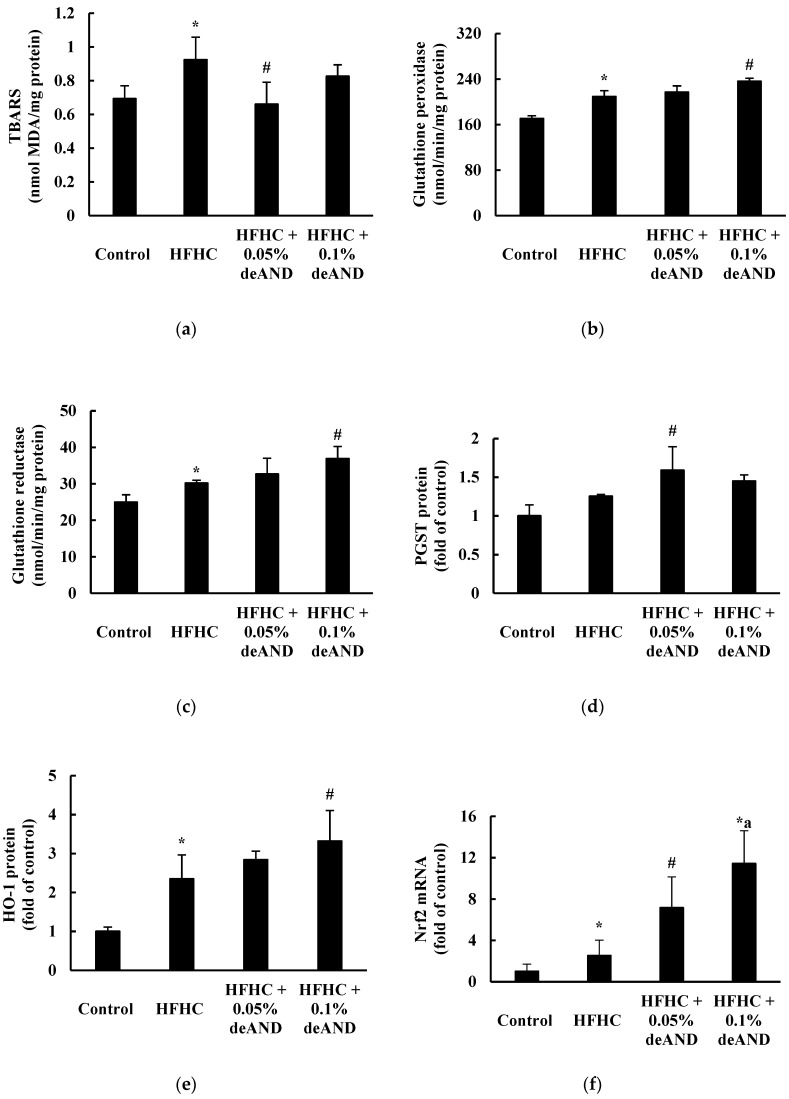
Effects of deAND supplementation on the hepatic TBARS content (**a**), GSH peroxidase activity (**b**), GSH reductase activity (**c**), pi form GSH-S-transferase (PGST) protein (**d**), HO-1 protein (**e**), and Nrf2 mRNA expression (**f**) in mice. Results are expressed as the mean ± S.D. (*n* = 6). * Significantly different from the Control group at *p* < 0.05. ^#^ Significantly different from HFHC group, *p* < 0.05. ^a^ Significantly different from the HFHC + 0.05% deAND group, *p* < 0.05.

Mice fed the HFHC diet for 11 weeks had increased (*p* < 0.05) hepatic TBARS, GSH-peroxidase and GSH reductase activities, as well as Nrf-2 mRNA and HO-1 protein expressions (Figure 6). These results suggest that mice fed the HFHC diet induced oxidative stress and stimulated Nrf-2-mediated downstream antioxidant enzyme activity and/or protein expression. Notably, deAND treatment reduced (*p* < 0.05) the hepatic TBARS level (especially in the 0.05% deAND group) and increased (*p* < 0.05) the GSH peroxidase and GSH reductase activities in liver. Significant increases (*p* < 0.05) in Nrf-2 mRNA and HO-1 protein expressions were observed after deAND treatment. These results indicate that deAND reduced oxidative stress by increasing Nrf-2-mediated downstream antioxidant enzyme activity and/or protein expression. 

### 3.8. Glutamate Levels in Plasma and the Liver

**Figure 7:**
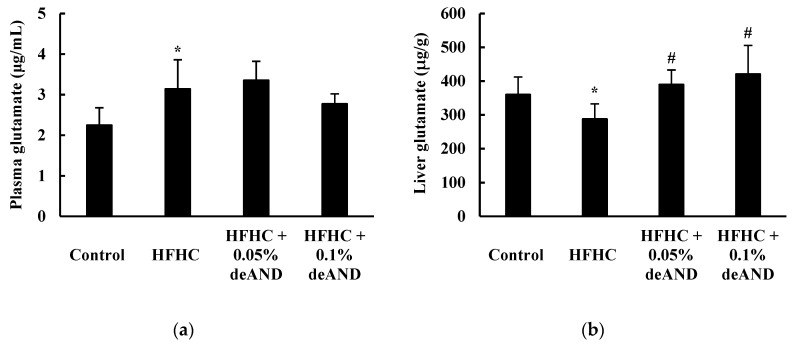
Effects of deAND supplementation on glutamate levels in the plasma (**a**) and liver (**b**) in mice. Results are expressed as the mean ± S.D. (*n* = 6). * Significantly different from the Control group, *p* < 0.05. ^#^ Significantly different from the HFHC group, *p* < 0.05.

As shown in Figure 7, mice fed the HFHC diet for 11 weeks had increased glutamate concentrations in the plasma (*p* < 0.05) but decreased in the liver (*p* < 0.05) when compared with animals fed the low-fat control diet. deAND treatment had no effect (*p* > 0.05) on plasma glutamate concentration, however, a higher (*p* < 0.05) glutamate content in liver was observed after the deAND treatment.

## 4. Discussion

In this study, deAND treatment reduced cholesterol accumulation, inflammation and lowered liver damage in mice fed a HFHC diet. In addition, deAND reduced NLRP3 inflammasome activation and oxidative stress in the liver. The Nrf-2-mediated downstream antioxidant enzyme activity and/or protein expressions in liver were upregulated by deAND. Although we could not distinguish whether the inflammation or oxidative stress in the liver induced by HFHC diet is derived from the hepatocytes or other immune cells, deAND treatment may ameliorate HFHC diet-induced steatohepatitis and liver damage possibly by increasing antioxidant and anti-inflammatory activities in the liver.

A previous study indicated that HFHC diet-induced histopathological changes in the livers of mice were accompanied by a significant accumulation of small cholesterol-containing droplets, which contained abundant cholesterol crystals [3] and free cholesterol [36]. The impact of dietary cholesterol was recently demonstrated to be a key factor in the transition from simple steatosis to NASH [37]. The accumulation of cholesterol crystals and free cholesterol in the liver may lead to a dysregulated cholesterol synthesis pathway and cause liver damage [3,38]. In this study, mice fed a HFHC diet for seven weeks showed morphological changes in the liver, alongside increased cholesterol accumulation and macrophage infiltration (Figure 2), and increased plasma ALT and AST activities. In addition, a dramatically reduced hepatic GSH content (–90.7%), lowered antioxidant enzyme activities (GSH peroxidase and GSH reductase), and elevated hepatic levels of TNF-α (+77.6%) and IL-1β (+55.6%) was observed in mice fed a HFHC diet. A higher apoptosis index (caspase 3/pro-caspase 3 ratio) was found in liver after HFHC feeding. These results indicate that mice fed the HFHC diet increased oxidative stress, inflammation, and liver damage. Mice treated with 0.1% deAND showed mildly lowered oxidative stress, as indicated by increasing GSH content and GSH reductase activity, and a significantly reduced inflammation by lowering TNF-α level in liver. Because increased oxidative stress, lipotoxicity, and inflammation play key roles in the progression of many fatty liver diseases [2], it is suggested that 0.1% deAND treatment may reduce HFHC diet-induced steatohepatitis and liver damage. 

To further investigate the exact mechanisms of deAND involved in antioxidant and anti-inflammatory activities associated with HFHC diet-induced oxidative stress and inflammation in liver, mice were fed a HFHC diet for a longer feeding time (11 weeks). The results showed that NLRP3 inflammasome activation was induced by feeding of the HFHC diet. The increased NLRP3 inflammasome activation can be stimulated by the accumulation of cholesterol and cholesterol crystals in the livers [3]. Shimada et al. also demonstrated that oxidized mitochondrial DNA can activate the NLRP3 inflammasome during apoptosis of cells [39]. In this study, deAND treatment not only reduced hepatic cholesterol accumulation, but also lowered apoptosis in the liver (Figure 3), factors that might lead to lower NLRP3 inflammasome activation as evidenced by attenuating caspase-1 mediated IL-1β release (Figure 5c,d). In addition to inhibition of caspase-1 activation, down regulation of the expressions of NLRP3, caspase-1, and IL-1β mRNA by deAND treatment (Figure 5e–f) further suggests that the anti-inflammatory property of this diterpenoid may act on transcriptional level by suppressing the activity of nuclear factor-kappa B (NFκB), which is responsible for expression of inflammatory cytokines [40]. These observations were similar to a recent study showing that AND could inhibit NLRP3 inflammasome activation and reduce inflammation in choline-amino acid-deficient diet-induced NASH [41].

Regarding oxidative stress and antioxidant activity in liver, mice fed the HFHC diet had higher hepatic lipid peroxide content and the activities of GSH peroxidase and GSH reductase. Notably, inductions of Nrf2 mRNA and HO-1 protein expressions were found in mice fed the HFHC diet, which may respond to cellular oxidative stress [42,43]. Therefore, it is suggested that long-term feeding with a HFHC diet (11 weeks) may increase oxidative stress in liver and, thus, trigger the expression of Nrf2-mediated downstream antioxidant enzymes to overcome the imbalance in the redox status [42,44]. In this study, deAND treatment lowered hepatic lipid peroxide and increased antioxidant enzyme activities could be explained by Nrf2 induction and, thereafter, lowered oxidative stress in the liver (Figure 6). It is known that a constant increase in lipotoxicity, followed by increases in oxidative stress and inflammation may promote the progress from NAFLD to NASH and impair liver function [2]. The present study is the first to demonstrate that deAND ameliorates steatohepatitis, liver fibrosis, and liver damage partially by enhancing hepatic Nrf2-mediated downstream antioxidant enzyme activities and suppressing NLRP3 inflammasome activation in HFHC diet-induced fatty liver disease.

It was noteworthy that supplementation of deAND in the HFHC diet also enhanced fecal excretions of cholesterol and total bile acids (Figure 4). Increased bile acid excretion, the major route of cholesterol degradation in liver, after deAND treatment might accelerate the biosynthesis of bile acid using cholesterol as the substrate and, thus, lower cholesterol level in liver. In addition, a lower bile acid reabsorption from small intestine into liver may contribute to a lower bile acid level in liver, and thus reduce dysregulated cholesterol metabolism and cholestatic liver injury [45]. Therefore, it is suggested that the enhancement of fecal excretions of cholesterol and total bile acids by deAND may lower bile acid and cholesterol contents in liver and, thus, reduce cholestatic liver injury in HFHC diet-induced steatohepatitis.

Metabolomics has been used as an effective diagnostic method to monitor specific metabolites produced by patients with hepatic steatosis and inflammation to allow early detection of liver disease [46]. Glutamate is an abundant free amino acid in various tissues, particularly the muscle and liver, which can act as a substrate or intermediate for various biochemical reactions and maintain health. It is one of the most important biomarkers for monitoring the status of NAFLD/NASH [46,47]. In this study, the plasma glutamate concentration increased in mice fed the HFHC diet, while the hepatic glutamate content decreased (Figure 7). A lower hepatic glutamate level is found in NAFLD/NASH patients [46]. Although the exact mechanism is unclear, the disturbance of glutamate homeostasis may play a role in the pathological changes in many diseases [48]. Therefore, a higher plasma glutamate concentration may be attributed from an increase in glutamate release from muscle or other tissues (e.g., liver) due to chronic inflammation in HFHC diet-induced fatty liver and liver injury in mice. In the present study, deAND increased glutamate level in the liver. This result suggests that deAND may improve fatty liver disease, at least in part, by increasing the hepatic glutamate level. Further study is warranted to clarify this finding.

In summary, the present study demonstrated that deAND may reduce steatohepatitis, liver fibrosis and liver injury, upregulate Nrf-2 triggered increase in proteins and/or activities of antioxidant enzymes, and lower inflammation by attenuating NLRP3 inflammasome activation in mice fed a HFHC diet. Therefore, deAND is likely to lower steatohepatitis and liver injury by increasing antioxidant and anti-inflammatory activities. 

## Figures and Tables

**Table 1:** Compositions of the experimental diets ^a^.

	Control	HFHC	HFHC + 0.05% deAND	HFHC + 0.1% deAND
Casein	20	20	20	20
Soybean oil	2.5	2.5	2.5	2.5
Lard	2	25	25	25
Sucrose	7	7	7	7
Corn starch	58.3	34.55	34.5	34.45
Cellulose	5	5	5	5
Choline chloride	0.2	0.2	0.2	0.2
Cholesterol		0.5	0.5	0.5
Cholic acid		0.25	0.25	0.25
AIN93 vitamin mixture ^a^	1	1	1	1
AIN93 mineral mixture	4	4	4	4
deAND			0.05	0.1
Total calories (Kcal/100 g diet)	381.7	494.9	494.7	494.5

^a^ AIN 93 vitamin and mineral mixtures were procured from ICN Biochemicals (Costa Mesa, CA, USA). HFHC, high-fat and high cholesterol. deAND, 14-Deoxy-11,12-didehydroandrographolide.

**Table 2:** Real-time PCR primers.

Gene	Forward Primer	Reverse Primer	Fragment Size
*Nlrp3*	5′-GAAGAAGAGAGGAGAGGAGGTCG-3′	5′-TTCACCAGTCTGGAAGAACAGGCAAC-3′	89
*Caspase 1*	5′-TTACTGCTATGGACAAGGCACG-3′	5′-GCTGATGGAGCTGATTGAAGCT-3′	142
*Il-1β*	5′-TTTGAAGAAGAGCCCTCCTC-3′	5′-AGGTGCTGATGTACCAGTTG-3′	436
*Nrf2*	5′- GATGACCATGAGTCGCTTGC-3′	5′- CCTGATGAGGGGCAGTGAAG-3′	73
*Gapdh*	5′-GCCTGGAGAAACCTGCCAAGTATG-3′	5′-GGGAGTTGCTGTTGAAGTCGCA-3′	213

**Table 3:** Plasma biochemical parameters ^a^.

	Control	HFHC	HFHC + 0.05% deAND	HFHC + 0.1% deAND
Total cholesterol (mg/dL)	125.5 ± 9.8	454.3 ± 38.2 ^*^	360.0 ± 52.0 ^#^	345.3 ± 86.1 ^#^
Triglyceride (mg/dL)	154.3 ± 24.3	97.7 ± 21.7 ^*^	113.5 ± 34.3	89.8 ± 14.0
ALT (U/L)	25.8 ± 12.6	232.8 ± 134.7 ^*^	44.0 ± 10.5 ^#^	49.4 ± 18.9 ^#^
AST (U/L)	90.3 ± 39.3	424.8 ± 173.6 ^*^	131.0 ± 70.3 ^#^	122.0 ± 43.2 ^#^
Hs-CRP (mg/dL)	0.28 ± 0.02	0.62 ± 0.14 ^*^	0.52 ± 0.03	0.56 ± 0.11
IL-1β (pg/mL)	2.95 ± 0.96	2.82 ± 1.07	1.99 ± 0.38	2.34 ± 0.69

^a^ Values are the mean ± SD, *n* = 5–6. Mice were fed the different experimental diets for 7 weeks. * Significantly different from Control group, *p* < 0.05. ^#^ Significantly different from HFHC group, *p* < 0.05. ALT: alanine aminotransferase; AST: aspartate aminotransferase; Hs-CRP: high-sensitive C reactive protein; IL-1β: interleukin-1β.

**Table 4:** GSH, GSH-related enzyme activities, lipid peroxidation, and inflammation in mice liver ^a^.

	Control	HFHC	HFHC + 0.05% deAND	HFHC + 0.1% deAND
GSH (nmol/mg protein)	2.81 ± 0.99	0.26 ± 0.03 ^*^	0.24 ± 0.05	0.32 ± 0.04 ^#^
TBARS (nmol MDA/mg protein)	0.79 ± 0.14	0.73 ± 0.14	0.76 ± 0.22	0.81 ± 0.14
GSH peroxidase (nmol/min/mg protein)	330.7 ± 62.0	283.3 ± 24.2	268.3 ± 72.3	243.2 ± 43.1
GSH reductase (nmol/min/mg protein)	49.6 ± 3.5	43.6 ± 2.9 ^*^	48.1 ± 5.8	50.6 ± 5.3 ^#^
GSH-S-transferase (nmol/min/mg protein)	317.7 ± 64.6	370.0 ± 39.9	297.2 ± 57.9	373.9 ± 86.7
TNF-α (pg/mg protein)	38.1 ± 7.5	67.7 ± 15.5 ^*^	54.4 ± 6.0	43.6 ± 12.0 ^#^
IL-1β (pg/mg protein)	0.04 ± 0.01	0.09 ± 0.06 ^*^	0.06 ± 0.01	0.05 ± 0.02

^a^ Values are the mean ± SD, *n* = 6. Mice were fed the different experimental diets for seven weeks. * Significantly different from Control group, *p* < 0.05. ^#^ Significantly different from HFHC group, *p* < 0.05. Abbreviations: GSH, Glutathione; TBARS, thiobarbituric acid–reactive substance; MDA, malondialdehyde; IL-1β, interleukin-1β; TNF-α, tumor necrosis factor-alpha.

## Supplementary Materials

The following are available online at https://www.mdpi.com/2072-6643/12/2/523/s1, Figure S1: Control group (a,e), HFHC group (b,f), HFHC + 0.05%deAND group (c,g), HFHC + 0.1%deAND group (d,h).
