# Supplementary material for: A Diterpenoid, 14-Deoxy-11, 12-Didehydroandrographolide, in Andrographis paniculata Reduces Steatohepatitis and Liver Injury in Mice Fed a High-Fat and High-Cholesterol Diet
Source: Nutrients. 2020 Feb 18;12(2):523. doi: 10.3390/nu12020523 (PMC7071475; doi:10.3390/nu12020523)
Supplement: Supplementary file 1 [file nutrients-12-00523-s001.pdf]

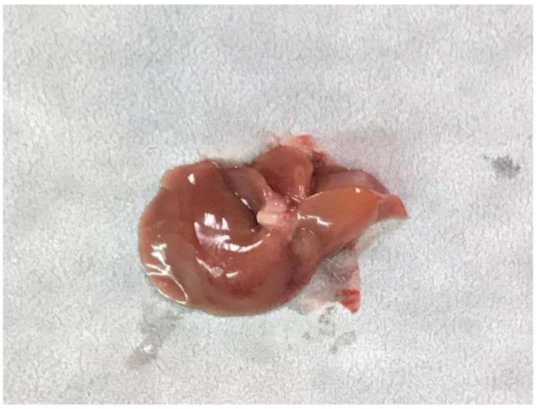

(**a**)

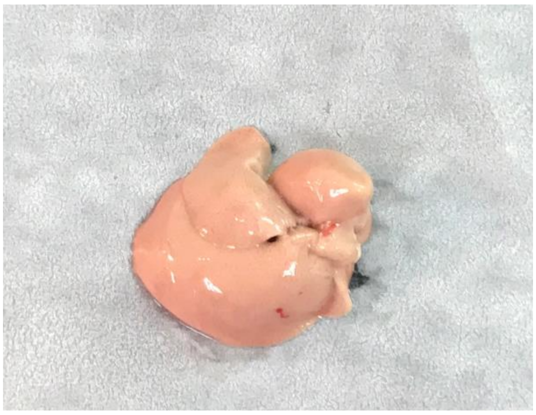

(b)

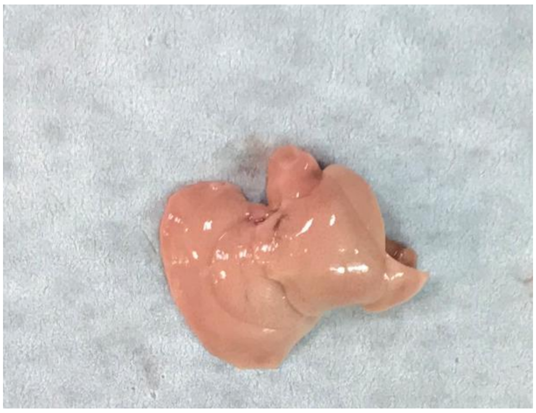

(c)

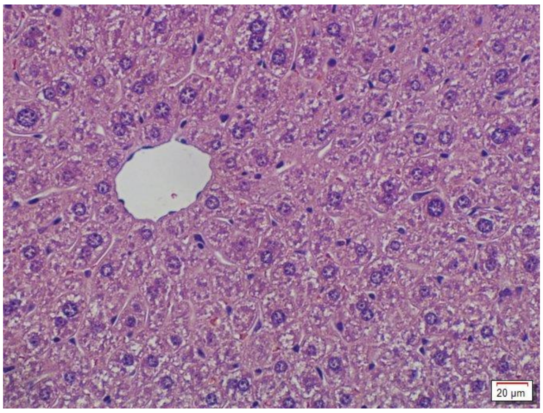

(**e**)

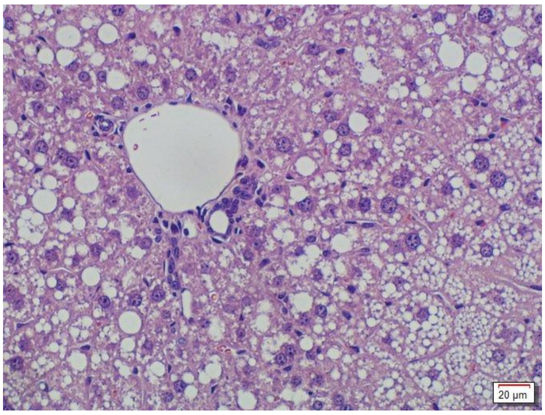

(f)

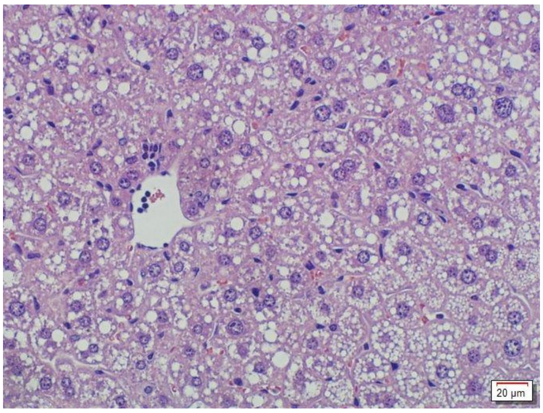

(g)

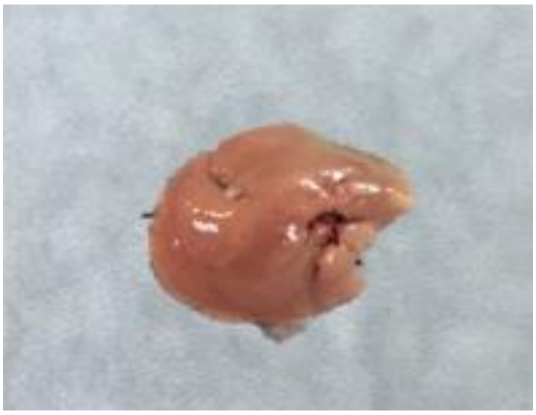

(d)

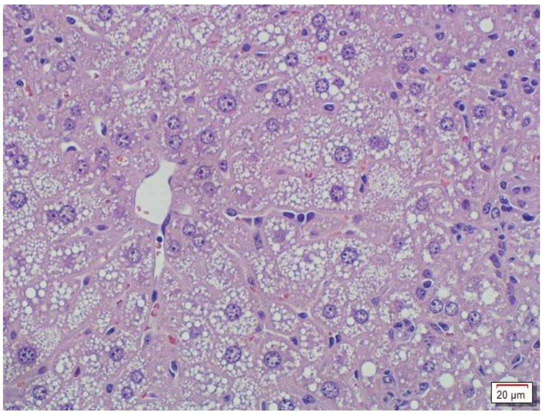

(h)

Figure S1. Control group (a,e), HFHC group (b,f), HFHC + 0.05%deAND group (c,g), HFHC + 0.1%deAND group (d,h).

In experiment II, mice fed the HFHC diet caused liver hypertrophy and increased fat accumulation (especially cholesterol) and perivenular inflammatory infiltrates in liver. deAND treatment for 11 weeks ameliorated these alternations caused by HFHC diet.
